# 9-*cis* β-Carotene Increased Cholesterol Efflux to HDL in Macrophages

**DOI:** 10.3390/nu8070435

**Published:** 2016-07-19

**Authors:** Sapir Bechor, Noa Zolberg Relevy, Ayelet Harari, Tal Almog, Yehuda Kamari, Ami Ben-Amotz, Dror Harats, Aviv Shaish

**Affiliations:** 1 The Bert W. Strassburger Lipid Center, Sheba Medical Center, Tel-Hashomer 5265601, Israel; sapirula@gmail.com (S.B.); noazolb@gmail.com (N.Z.R.); harari.ayelet1@gmail.com (A.H.); tal.almog@sheba.health.gov.il (T.A.); Yehuda.Kamari@sheba.health.gov.il (Y.K.); Dror.Harats@sheba.health.gov.il (D.H.); 2 Sackler School of Medicine, Tel-Aviv University, Tel Aviv 6997801, Israel; 3 N.B.T., Eilat 8810602, Israel; amiba@bezeqint.net

**Keywords:** 9-*cis*-β-carotene, reverse cholesterol transport, cholesterol efflux, ATP Binding Cassette transporter A1, ATP Binding Cassette transporter G1, apolipoprotein E, retinoic x receptor

## Abstract

Cholesterol efflux from macrophages is a key process in reverse cholesterol transport and, therefore, might inhibit atherogenesis. 9-*cis*-β-carotene (9-*cis*-βc) is a precursor for 9-*cis*-retinoic-acid (9-*cis*-RA), which regulates macrophage cholesterol efflux. Our objective was to assess whether 9-*cis*-βc increases macrophage cholesterol efflux and induces the expression of cholesterol transporters. Enrichment of a mouse diet with βc from the alga *Dunaliella* led to βc accumulation in peritoneal macrophages. 9-*cis*-βc increased the mRNA levels of CYP26B1, an enzyme that regulates RA cellular levels, indicating the formation of RA from βc in RAW264.7 macrophages. Furthermore, 9-*cis*-βc, as well as all-*trans*-βc, significantly increased cholesterol efflux to high-density lipoprotein (HDL) by 50% in RAW264.7 macrophages. Likewise, food fortification with 9-*cis*-βc augmented cholesterol efflux from macrophages ex vivo. 9-*cis*-βc increased both the mRNA and protein levels of ABCA1 and apolipoprotein E (APOE) and the mRNA level of ABCG1. Our study shows, for the first time, that 9-*cis*-βc from the diet accumulates in peritoneal macrophages and increases cholesterol efflux to HDL. These effects might be ascribed to transcriptional induction of ABCA1, ABCG1, and APOE. These results highlight the beneficial effect of βc in inhibition of atherosclerosis by improving cholesterol efflux from macrophages.

## 1. Introduction

Atherosclerosis is a chronic inflammatory disease caused by the interaction between oxidized lipoproteins and cells in the arterial wall, especially macrophages [1]. Ingestion of modified lipoproteins by macrophages leads to the formation of macrophage-derived foam cells, an early cellular hallmark of atherosclerotic plaque development and lesion progression. Reverse cholesterol transport (RCT) is a process in which cholesterol accumulated in peripheral cells is transported by high-density lipoprotein (HDL) to the liver for excretion, preventing the accumulation of excess cholesterol and foam cell formation. Cholesterol efflux from macrophage to HDL is a key process in RCT and, therefore, might inhibit atherosclerosis formation and progression [2].

Cholesterol export from macrophages to lipoproteins occurs by passive aqueous diffusion or by active transport via the ATP-binding cassette transporter superfamily (ABCs), predominantly, ABCA1 and ABCG1, and by scavenger receptor class B, type I (SR-BI) [3]. ABCA1 facilitates cholesterol efflux to apolipoprotein A-I (APOA-I) and apolipoprotein E (APOE) to form the nascent HDLs, followed by further efflux via ABCG1 to create mature HDL particles [4]. ABCA1 or ABCG1 knockout macrophages and mice show reduced cholesterol efflux [5,6]. Aside from the fact that ABCA1 mediates cholesterol efflux to APOE, APOE’s involvement in RCT is unclear. Recent studies showed that macrophages, but not systemic APOE, are necessary for macrophage cholesterol efflux in vivo, probably by increasing cholesterol availability to different acceptors [7].

Retinoids (vitamin A derivatives) regulate many biological activities, such as embryo development and cell proliferation, differentiation, and apoptosis [8] by activation of the nuclear receptors retinoid x receptor (RXR) and retinoic acid receptor (RAR) [9]. While RAR is activated by all-*trans*-retinoic acid (ATRA), and 9-*cis*-retinoic acid (9-*cis*-RA), RXR could be activated by 9-*cis*-RA, it is not clear whether this is, indeed, its endogenous ligand [10]. In macrophages, ligands of RXR and RAR regulate lipid metabolism and cholesterol efflux by forming heterodimers with other nuclear receptors [11]. ATRA and RAR agonists induce ABCA1 and ABCG1 expression and increase cholesterol efflux to APOA-I and HDL [12,13]. Correspondingly, 9-*cis*-RA and rexinoids (synthetic retinoids) induce ABCA1, ABCG1, and APOE expression in macrophages [14,15] and enhance cholesterol efflux to APOA-I and HDL [16,17].

Carotenoids are a source for retinoids, among them RA, and, therefore, might increase macrophage cholesterol efflux. Despite the potential of carotenoids, only a few works have examined their effect on macrophage cholesterol efflux in vitro. Moreover, the effect of carotenoids and retinoids on RCT in vivo has not been tested. Although in a recent report, all-*trans*-βc had no effect on ABCA1, ABCG1, and APOE mRNA levels, compared to 9-*cis*-RA and ATRA [15], the carotenoid astaxanthin enhanced macrophage cholesterol efflux to HDL and APOA-I and increased ABCA1 and ABCG1 mRNA levels, but high doses were required to achieve this effect [18]. Recently, we have shown that 9-*cis*-βc inhibits foam cell formation in the RAW264.7 cell line, similar to 9-*cis-*RA [19]. This might be due to inhibition of cholesterol uptake and/or enhancement of cholesterol efflux.

Several epidemiological studies showed an association between consumption of βc and decreased cardiovascular disease incidence [20,21], while all-*trans*-βc is a source for ATRA, 9-*cis*-βc cleavage produced 9-*cis*-RA and ATRA [22]. The 9-*cis*-βc is found in lower levels than the all-*trans* isomer in fruits and vegetables consumed in our diet, and it is present in the highest known levels in the unicellular, halo-tolerant alga *Dunaliella bardawil* [23]. We have previously reported the favorable effects of a 9-*cis*-βc-rich diet, including elevation of plasma HDL-cholesterol in fibrate-treated patients [24], inhibition of atherogenesis and fatty liver development in LDLR–/– mice [25], and prevention of atherosclerosis progression in high-fat diet-fed APOE–/– mice [26]. Nevertheless, the anti-atherosclerotic mechanism of *Dunaliella* has not yet been determined. Thus, in the current study, we sought to investigate whether exposure of macrophages to carotenoids enhances cholesterol efflux to HDL.

## 2. Materials and Methods

### 2.1. Animals

Twelve-week-old male LDL receptor knockout mice (LDLR–/–) (C57BL/6 background) were obtained from Jackson Laboratories. The animals were caged in an animal room with alternating 12-h periods of light and dark with free access to feed and water. Mice were killed with isoflurane. All protocols were approved by the Animal Care and Use committee of Sheba Medical Center, Tel-Hashomer (833/13).

### 2.2. Diet

Mice were fed a chow diet (18% protein, 5% fat; TD2018, Harlan Teklad, Houston, TX, USA). To enrich the diet with βc, we used the powder of the alga *Dunaliella bardawil* containing 6% βc (weight/weight), comprised of 50% all-*trans* and 50% 9-*cis* isomers [27] (a gift from Nikken Sohonsha, Gifu, Japan). To prepare the feed, 0.75 L of distilled hot water was mixed with 28 g of gelatin until the solution was clear. Then, 1 kg of powdered feed and *Dunaliella* powder (80 g/kg feed, containing 3 g/Kg all-*trans*-βc and 3 g 9-*cis*-βc) was thoroughly mixed with the warm gelatin solution. After solidification, the feed was divided into tablets and stored at −20 °C in the freezer; the feed was replaced every other day to minimize the oxidation and degradation of its ingredients.

### 2.3. Study Design

The aim of the ex vivo assay was to study whether *Dunaliella* powder would affect cholesterol efflux to HDL. We also performed an in vitro assay that allowed us to examine the direct effect of specific carotenoids and retinoids on cholesterol efflux to HDL in macrophages. Ten 12-week-old male LDLR–/– mice were allocated into two groups, five animals per group. The control group was fed a regular diet (chow) with no supplementations. The *Dunaliella* group was fed the diet fortified with algal powder. After four weeks of treatment, the mice received peritoneal injections with thioglycolate, followed by macrophage isolation.

### 2.4. Tissue Culture

RAW264.7, mouse macrophage cell line, was maintained in Dulbecco’s Modified Eagle Medium (DMEM) 4.5 g/L glucose containing 10% FBS (Biological Industries Ltd., Kibbutz Beit-Haemek, Israel), 50 U/mL penicillin, and 50 µg/mL streptomycin enriched with 2 mM glutamine, purchased from ATCC (TIB-71, Rockville, MD, USA). 1 × 10^6^ cells/well were plated into six-well plates for gene expression and Western blot analysis, and 1 × 10^5^ cells/well were plated into 24-well plates for cholesterol efflux experiments. Forty-eight hours post-seeding, the medium was replaced and cells were incubated for 24 h in serum-free DMEM containing 2 µM βc. βc was dissolved in hexane, and the concentration was then determined by 450 nm absorbance, followed by the addition of TWEEN 40 in acetone (1:4 respectively) to a total concentration of 0.1% tween in the cell medium. Solvents were evaporated and the residue was solubilized in the medium. βc had no-toxic effect in these concentration [28,29,30,31] and βc toxicity has not been examined since microscopic examination showed no differences between βc-treated and control cells. 

Mouse peritoneal macrophages were recovered three days after thioglycolate injection into the peritoneum and isolated as described previously [32]. 5 × 10^4^ cells/well were plated into 24-well plates in DMEM supplemented with 10% FCS for cholesterol efflux analysis. Cells were allowed to adhere to the plates for 6 h, were washed with PBS, and were then replaced to serum free DMEM with 0.5 µCi/mL ^3^H-cholesterol (PerkinElmer, Waltham, MA, USA) for 18 h. For carotenoids extraction, cells were collected, counted, and stored at −80 °C until carotenoid extraction.

### 2.5. βc Analysis

Peritoneal macrophages were extracted with 2 mL of ethanol containing 10 µM butylated hydroxytoluene which was followed by the addition of 2 mL hexane and 1 mL of Double-distilled water (DDW). The samples were mixed and centrifuged for 5 min at 1000× *g*. The hexane layer was separated and dried under a stream of N2. Dried samples were suspended in 100 µL hexane, and βc concentrations were determined by reverse phase HPLC on a YMC C30 column (CT995031546QT, 150 × 4.6, 3 μm particle size; YMC Inc., Allentown, PA, USA) with methanol/methyl-tert-butyl-ether/water 1.5% ammonium acetate as the mobile phase at a flow rate of 1 mL/min [33]. βc was detected by monitoring its absorbance at 450 nm and by comparison with the retention times of authentic standards. Results are expressed as nanogram of βc per 10^6^ cells seeded.

### 2.6. HDL Preparation

Ultracentrifugation is a common and acceptable method to isolate HDL for cholesterol efflux assessment. This method had been used to examine the effect of different carotenoids and retinoids on cholesterol efflux by several research groups [13,17,34]. In our study, HDL was obtained from healthy volunteers by sequential ultracentrifugation (density 1.125–1.210 g/mL) [35] and its concentration was determined using the Lowry method [36].

The Helsinki committee of Sheba Medical Center approved all procedures (1340-14-SMC) and the research was conducted with full exemption from informed consent. Existing plasma samples were pooled and used in the experiments with no identifiers linking individuals to the samples.

### 2.7. Cholesterol Efflux Assays

For in vitro experiments, after incubation with βc, RAW264.7 cells were washed three times with PBS and radiolabeled for 6 h with serum-free DMEM containing 0.5 µCi/mL ^3^H-cholesterol. The cells were washed three times with PBS and incubated for 0.5 h in the presence or absence of serum-free DMEM containing 1 µg/mL HDL. The media was then collected and centrifuged to remove debris, and cells were washed four times with PBS and lysed by incubation for 1 h in 60 °C with 1 M NaOH. After determination of the radioactivity in the medium and cell lysate by liquid scintillation counting, cholesterol efflux was calculated as the percentage of the radioactivity removed to the media over the total radioactivity (cells plus media) after the subtraction of the non-specific HDL-free media [37]. In ex vivo experiments, after 18 h of radiolabeling with 0.5 µCi/mL ^3^H-cholesterol, cells were washed three times with PBS and incubated for 0.5 h in the presence or absence of serum-free DMEM containing 1 µg/mL HDL. Cholesterol efflux was determined as mentioned above. All cholesterol efflux assays were performed in triplicate. The 0.5 h efflux time point was chosen after calibration expirements. 

### 2.8. RNA Extraction and Real-Time PCR

Total RNA was extracted using a Nucleospin RNA II Kit according to the manufacturer’s instructions (Macherey-Nagel, Düren, Germany). RNA was reverse-transcribed using a High-Capacity cDNA Reverse Transcription Kit according to the manufacturer’s instructions (Applied Biosystems, Bleiswijk, Nederland). Quantitative real-time PCR was performed with a 7900HT PCR machine (Applied Biosystems), FastStart Universal Probe Master ROX (Roche, Mannheim, Germany), and a FAM-labeled TaqMan primer and probe (Roche) for mouse CYP26B1 (316002, Roche), ABCA1 (300801, Roche), ABCG1 (300805, Roche), ABCG4 (300813, Roche), and APOE (310773, Roche). We used *GAPDH* (307884, Roche) as a reference gene.

### 2.9. Western Blot Analysis

At the end of incubation with βc, cells were lysed in RIPA buffer (R0278, Sigma, St. Louis, MO, USA). The lysates were incubated on ice for 30 min and were centrifuged for 20 min at 4 °C at 13,000× *g*, and the supernatants were then taken. Protein concentrations were determined by a Pierce BCA Protein Assay Kit (Thermo Scientific, Waltham, MA, USA). Cell lysates were added with 4X Laemmli sample buffer, and 40 µg proteins were separated on a 7% SDS polyacrylamide gel (ABCA1 and ABCG1) or boiled and separated by 12% SDS polyacrylamide gel (APOE). Proteins were transferred to nitrocellulose membranes. After non-specific blocking with skim milk (ABCA1) or BSA (ABCG1 and APOE) for 1.5 h, the membranes were incubated with anti-ABCA1 (NB400-105, Novus Biologicals, Littleton, CO, USA), anti-ABCG1 (sc-11150, Santa Cruz, Santa Cruz, CA, USA), or anti-APOE (ab1906, Abcam, Cambridge, MA, USA) overnight at 4 °C. The membranes were then washed three times with Tris-buffered saline added with 0.1% Tween 20 (TBST), and then incubated with an appropriate horseradish peroxidase-conjugated secondary antibody. Membranes were washed three times with TBST, incubated with an enhanced chemiluminescence solution (Pierce), and exposed to X-ray films. Bands were quantified by densitometry and normalized to those of β-actin (Santa Cruz).

### 2.10. Statistical Analysis

Data were reported as mean ± standard error of the mean. The in vitro studies results were analyzed by one-way analysis of variance (ANOVA) and the post-hoc Tukey method. Student’s *t*-test was used to compare ex vivo cholesterol efflux. All statistical analyses were performed with SPSS 22.0 software (IBM Corporation, Armonk, NY, USA). Statistical significance was obtained when *p* values were less than 0.05.

## 3. Results

### 3.1. β-Carotene-Enriched Diet Led to 9-cis-βc and All-Trans-βc Accumulation in Mouse Peritoneal Macrophages 

**Figure 1:**
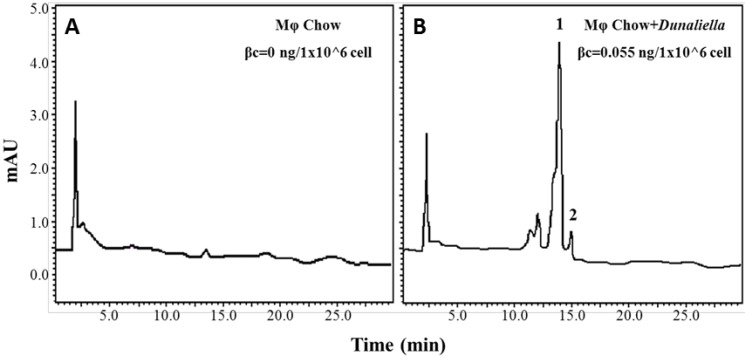
Increased accumulation of carotenoids in peritoneal macrophages isolated from mice fed with a βc rich diet. High-Performance Liquid Chromatography (HPLC) chromatograms of carotenoids in peritoneal macrophages isolated from LDLR–/– mice fed chow diet for four weeks in the absence (**A**) or presence of *Dunaliella* (**B**). 1-all-*trans*-βc, 2-9-*cis*-βc. Separation was conducted on C30 column and detection at 450 nm. φ, macrophages.

We hypothesized that 9-*cis*-βc increases the process of macrophage cholesterol efflux, so we first sought to study whether dietary βc is delivered to macrophages. Therefore, we fed LDLR–/– mice with a chow diet fortified with *Dunaliella* powder for four weeks and determined the βc content in the peritoneal macrophages. In macrophages isolated from *Dunaliella*-fed mice, βc levels were dramatically higher, 0.55 ng/1 × 10^6^ cells (Figure 1B), than macrophages from the control group, whose carotenoids content was below the detection level (Figure 1A). Although the *Dunaliella* powder contained similar levels of all-*trans* and 9-*cis* isomers, the macrophages contained higher levels of all-*trans*-βc than 9-*cis*-βc.

### 3.2. 9-cis-βc Induced Cytochrome P450 26B1 (CYP26B1) Expression

**Figure 2:**
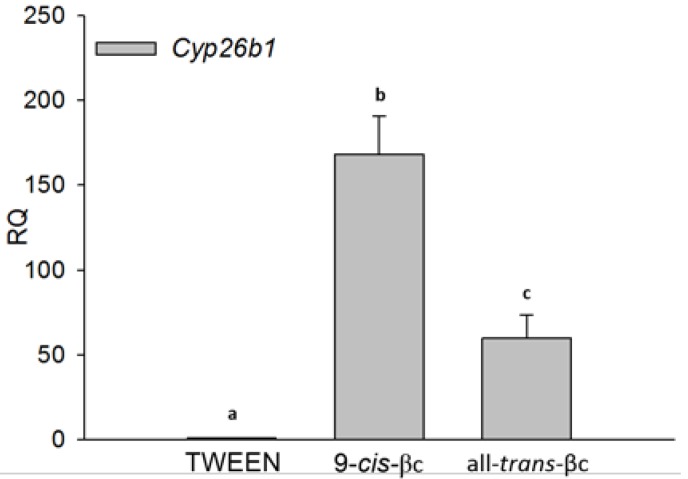
9-*cis*-βc induced macrophage cytochrome P450 26B1 (CYP26B1) expression. RAW264.7 macrophage cells were treated for 24 h with vehicle (TWEEN 40), 2 µM of 9-*cis*-βc or all-*trans*-βc. The expression of CYP26B1 mRNA was measured by quantitate real-time PCR assays (TaqMan) standardized against GAPDH mRNA levels. The results are expressed as mean ± SE. of six independent experiments. Different letters represent significant differences between treatments, *p* < 0.05.

To investigate whether βc can regulate cholesterol efflux through its conversion to RA, we examined CYP26B1 expression levels. CYP26 enzymes family tightly regulates RA levels [38]. Due to the low levels of RA and difficulty in determining RA cellular levels, CYP26B1 has been used as an indicator for RA levels [39,40,41]. In RAW264.7 macrophages, 9-*cis*-βc and all-*trans*-βc significantly increased CYP26B1 mRNA levels by 168 and 60 fold, respectively (Figure 2).

### 3.3. 9-cis-βc Increased Macrophage Cholesterol Efflux to HDL in Vitro and ex Vivo

**Figure 3:**
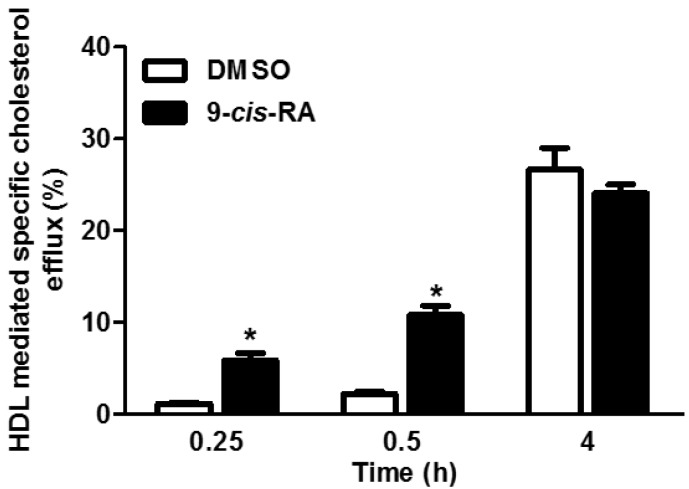
Calibration of different incubation periods with High-Density Lipoprotein (HDL). Peritoneal macrophages isolated from C57BL/6 mice were treated for 24 h with or without vehicle (DMSO) or 2 µM of 9-*cis*-retinoic acid (RA). Cells were labeled with 0.5 µCi/mL ^3^H-cholesterol in serum-free Dulbecco’s Modified Eagle Medium (DMEM). Cholesterol efflux was determined after 0.5, 1, and 4 h incubation with 1 µg/mL HDL. The results are expressed as mean ± SE of one experiment performed in triplicate.* *p* < 0.05.

As demonstrated in Figure 3, 9-*cis*-RA significantly increased cholesterol efflux to HDL when the efflux period was 0.25 or 0.5 h. The effect was abolished after a longer incubation period with the acceptor. Based on these results, we examined the effect of carotenoids on cholesterol efflux after 0.5 h incubation with HDL. In addition, a long incubation time without treatment could result in reduced expression of the cholesterol transporters and obscure the beneficial effect in the short incubation time. Moreover, long efflux periods increase the contribution of non-specific efflux and random diffusion [42]. 

**Figure 4:**
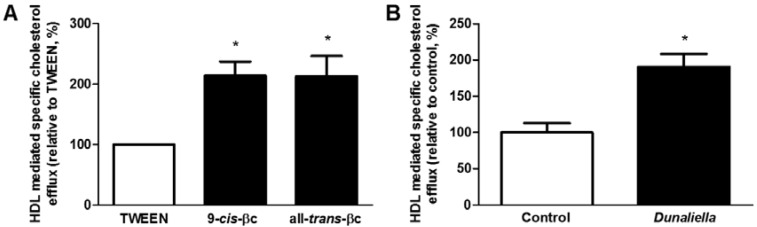
9-*cis*-βc increased macrophage cholesterol efflux to HDL. (**A**) RAW264.7 macrophages were treated for 24 h with vehicle (TWEEN 40), 2 µM of 9-*cis*-βc or all-*trans*-βc. Cells were labeled with serum-free DMEM containing 0.5 µCi/mL ^3^H-cholesterol for 6 h. Cholesterol efflux was determined after 0.5-h incubation with 1 µg/mL HDL, relative to TWEEN (2% efflux). The results are expressed as mean ± SE of five independent experiments, each preformed in triplicate. * *p* < 0.05; (**B**) Peritoneal macrophages isolated from LDLR–/– mice fed chow diet fortified with (*n* = 5) or without (*n* = 5) *Dunaliella* for four weeks. Cells were labeled with serum-free DMEM containing 0.5 µCi/mL ^3^H-cholesterol for 18 h. Cholesterol efflux was determined after 0.5-h incubation with 1 µg/mL HDL. The results are expressed as mean ± SE. * *p* < 0.05.

Following the demonstration that dietary βc is accumulated in macrophages, we investigated whether 9-*cis*-βc, isolated from *Dunaliella*, accelerates cholesterol efflux from RAW264.7 macrophages to HDL. Pre-incubation with 2 µM of 9-*cis*-βc or all-*trans*-βc significantly increased cholesterol efflux to HDL by 214% and 213%, respectively (*p* < 0.05) (Figure 4A). Moreover, cholesterol efflux from macrophages isolated from mice fed with 9-*cis*-βc-rich *Dunaliella* powder was higher than cholesterol efflux from macrophages isolated from control mice (*p* < 0.05) (Figure 4B).

### 3.4. 9-cis-βc Induced mRNA and Protein Levels of Genes Involved in Macrophage Cholesterol Efflux

**Figure 5:**
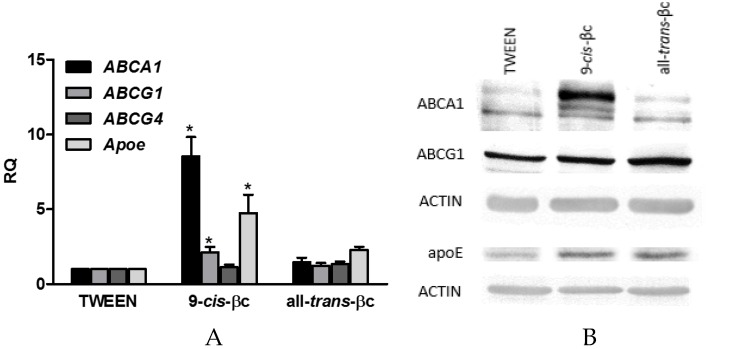
9-*cis*-βc-induced mRNA and protein expression levels of genes involved in cholesterol efflux in macrophages. RAW264.7 macrophage cells were treated for 24 h with vehicle (TWEEN 40), 2 µM of 9-*cis*-βc or all-*trans*-βc. (**A**) The expression of ABCA1, ABCG1, ABCG4, and APOE mRNA were measured by quantitate real-time PCR assays (TaqMan) standardized against GAPDH mRNA levels. The results are expressed as mean ± SE. of six independent experiments. * *p* < 0.05; (**B**) ABCA1, ABCG1, and APOE protein levels were determined by Western blot analysis. The results represent one of five independent experiments.

To determine whether the increase in cholesterol efflux can be due to the induction of known genes involved in this process, RAW264.7 cells were incubated for 24 h with 2 µM of 9-*cis*-βc or all-*trans*-βc. We selected ABCA1, ABCG1, and APOE, since these are target genes for RXR heterodimers in macrophages, and they are critically involved in cholesterol efflux [43,44,45,46,47,48]. 9-*cis*-βc increased the mRNA levels of ABCA1, ABCG1, and APOE by 8.5-, 2.2-, and 4.8-fold, respectively (Figure 5A). In contrast, all-*trans*-βc had no effect on ABCA1, ABCG1, and APOE mRNA levels (Figure 5A). We further investigated the expression of ABCG4, a member in the ABCG family that forms heterodimers with ABCG1. ABCG4 is mainly expressed in the brain and its expression in macrophages is controversial [49,50]. We found that ABCG4 is expressed in macrophages, but βc isomers did not affect its expression (Figure 5A). Similarly, both βc isomers had no effect on SR-BI mRNA levels (data not shown). 

In accordance with the effect on mRNA levels, 9-*cis*-βc increased ABCA1 protein levels by 2.1 fold and all-*trans*-βc increased it by only 1.3-fold (Figure 5B). There was no significant induction in ABCG1 protein levels after incubation with the βc isomers (Figure 5B). The strongest induction was observed in APOE protein levels. 9-*cis*-βc increased APOE protein levels by 2.8-fold and all-*trans*-βc by 3.6-fold (Figure 5B).

## 4. Discussion

Cholesterol efflux plays a critical role in reducing the accumulation of cholesterol in macrophages and, therefore, in the inhibition of atherosclerotic plaque development and progression. Recent studies in our laboratory demonstrated the athero-protective effects of 9-*cis*-βc-rich alga *Dunaliella bardawil* in LDLR–/– and APOE–/– mice [25,26] and their inhibitory effect on foam cell formation [19]. In this study, we investigated whether 9-*cis*-βc increases macrophage cholesterol efflux. We showed that: (I) dietary 9-*cis*-βc is accumulated in macrophages; (II) 9-*cis*-βc induces the expression of the cholesterol transporters ABCA1 and ABCG1 and macrophage APOE; and (III) 9-*cis*-βc significantly increases macrophage cholesterol efflux to HDL.

We have found that a diet fortified with βc from the alga *Dunaliella bardawil* leads to the accumulation of 9-*cis*-βc and all-*trans*-βc in peritoneal macrophages. There are no previous reports in the literature showing that dietary βc is accumulated in macrophages. However, several findings have hinted that βc is present in macrophages: (I) βc is found in LDL, the major vehicle of intact carotenoids in human plasma [51]. As macrophages engulf LDL, it is reasonable that βc delivered by LDL is transferred to macrophages and then accumulated in these cells; (II) the presence of carotenoids in atherosclerotic plaques was documented more than 50 years ago, and the yellow hue of human atherosclerotic lesions is mainly due to the accumulation of several carotenoids [52]; and (III) it has been demonstrated that βc activates nuclear receptors, RXR and RAR, in the aorta of APOE–/– mice, suggesting the arrival of βc to the vessel wall [53]. Numerous studies, including studies provided βc from *Dunaliella bardawil*, have shown increased levels of βc after food fortification with βc in various tissues [54,55]. Similar to our finding, all-*trans*-βc levels were higher than 9-*cis*-βc levels [56]. The detection of both all-*trans*-βc and 9-*cis*-βc in macrophages supports our hypothesis that dietary βc has the potential to increase macrophage cholesterol efflux.

Indeed, macrophages isolated from mice fed with 9-*cis*-βc rich *Dunaliella* powder had greater cholesterol efflux capacity. Since the alga contains both 9-*cis*-βc and all-*trans*-βc, it is impossible to conclude from such treatment which carotenoid is the more potent. In addition, the conversion of these carotenoids to different retinoids in the body complicates the situation even more. However, the results suggest that dietary βc might affect macrophage cholesterol efflux.

Since CYP26B1 expression, an indicator of RA levels, increased following βc treatment, it is reasonable to hypothesize that this effect is due to βc conversion to RA, or another retinoid [10]. Although, several studies have demonstrated that RA induces macrophage cholesterol efflux to HDL and APOA-I [13,17,34], βc’s effect on this process has not been addressed. Yet, the carotenoid astaxanthin induced macrophage cholesterol efflux and both ABCA1 and ABCG1 expression in RAW264.7 cells; however, very high doses were required to attain this effect [18]. Taken together, these data and our results support the hypothesis that 9-*cis*-βc induces macrophage cholesterol efflux to HDL via its conversion to RA. Nevertheless, we do not rule out the possibility that βc itself can also function as a modulator of cholesterol efflux in macrophages.

Cholesterol efflux is mediated mainly by ABCA1 and ABCG1, while recent work demonstrates a significant role of intracellular APOE production in this process [7]. This gene's regulation is mediated by RXR heterodimers; therefore, we sought to study whether they are induced by 9-*cis*-βc treatment. We have found association between augmented macrophage cholesterol efflux to HDL following 9-*cis*-βc administration, and increased expression in ABCA1, ABCG1, and APOE mRNA and protein levels. This is in agreement with the induction of ABCA1, ABCG1, and APOE in astrocytes treated with βc [31]. Similar to βc, the carotenoid β-cryptoxanthin increased ABCA1 and ABCG1 mRNA levels and ABCA1 protein levels in peritoneal macrophages. The researchers claimed that β-cryptoxanthin, contrary to our assumption, activates RAR as a whole molecule without its conversion to RA [57]. Although lycopene, a carotenoid found primarily in tomatoes, is not a pro-vitamin A, it increased the protein levels of ABCA1 in THP-1 cells [58]. Hence, these reports contribute to our finding showing upregulation in expression of cholesterol transporters by 9-*cis*-βc. 

Even though the increase in cholesterol efflux by all-*trans*-βc did not involve ABCA1 and ABCG1 induction, it dramatically increased APOE protein levels. In agreement, in THP-1 macrophages, all-*trans*-βc did not affect transporters expression, while 9-*cis*-RA and ATRA induced it [30]. Therefore, we assume that although all-*trans*-βc had no effect on ABCA1 and ABCG1 expression, the increase in APOE levels is sufficient to accelerate cholesterol efflux to HDL in our experimental conditions. However, we cannot rule out the possibility that βc accelerated macrophage cholesterol efflux by other mechanisms, unrelated to the modulation of ABCAs or APOE expression. Nevertheless, some might argue that the minor induction in ABCA1 levels by all-*trans*-βc might be enough for increasing cholesterol efflux. 

The different effects of 9-*cis*-βc and all-*trans*-βc on gene expression suggest different mechanisms by which the two isomers increase macrophage cholesterol efflux. In order to further understand the effect of βc on macrophage cholesterol efflux we examined the expression of ABCG4. ABCG1/4 heterodimers mainly regulate cholesterol efflux in the brain, but not in macrophages [49,50]. Our results indicated that macrophages express ABCG4, but carotenoids do not increase its expression levels. While Engel et al. showed that combined treatment with oxysterols and retinoids induced ABCG4 expression [41], Tarr et al. demonstrated that, unlike ABCA1 and ABCG1, ABCG4 is differentially regulated [59]. In addition to ABCG1 and ABCG4, SR-BI also facilitates cholesterol efflux to HDL [60] but has minor contributions to macrophage cholesterol efflux [42] and βc isomers did not affect its expression (data not shown). Further studies are needed in order to clarify the mechanism by which βc isomers differentially augmented cholesterol efflux. 

The finding that the RA degradation enzyme CYP26B1 is expressed in human atherosclerotic lesions led to the assumption that ATRA levels are regulated locally in the vessel wall [61]. Thus, carotenoids and retinoids might affect atherosclerosis progression by regulating macrophage cholesterol accumulation and atherosclerotic inflammatory response. Recent study has shown that ATRA, retinol, and all-*trans*-βc enhanced macrophages phagocytosis [62]. Moreover, βc reduced inflammatory markers [63] and the production of interleukin-1 β (IL-1β), IL-6, tumor necrosis factor α (TNF-α), and monocyte chemotactic protein-1 (MCP-1) in macrophages [30,63]. These results raise the question whether βc regulates macrophage polarization and preferentially M2 polarization. M2 macrophages produce anti-inflammatory cytokines and express ABCA1 [64] and, hence, has the potential to induce macrophage cholesterol efflux.

Based on the results of the present study, we propose the following model for macrophage cholesterol efflux induction by 9-*cis*-βc: (I) dietary 9-*cis*-βc is accumulated in macrophages; (II) the conversion of βc to active metabolites in macrophages induces the expression of the cholesterol transporters ABCA1 and ABCG1, and macrophage APOE; and (III) this induction increases macrophage cholesterol efflux to HDL. The identification of the key βc metabolites involved in enhancing cholesterol efflux from macrophages to HDL is currently under investigation in our laboratory. 

## 5. Conclusions 

In conclusion, our findings suggest that dietary 9-*cis*-βc delivered to macrophages in the vessel wall has the potential to enhance cholesterol efflux from lesion macrophages and, consequently, to inhibit atherogenesis.

## Abbreviations

The following abbreviations are used in this manuscript:

9-*cis*-βc	9-*cis*-β-carotene
RA	Retinoic Acid
ABCA1	ATP Binding Cassette transporter A1
ABCG1	ATP Binding Cassette transporter G1
APOE	Apolipoprotein E
RXR	Retinoid X Receptor
